# An Extended Follow-Up on Blood Pressure in a Patient With New-Onset Essential Hypertension: Early-Morning Home, Morning Home, and Office Readings

**DOI:** 10.7759/cureus.52520

**Published:** 2024-01-18

**Authors:** Yukihito Higashi, Shinji Kishimoto

**Affiliations:** 1 Department of Regenerative Medicine, Research Institute for Radiation Biology and Medicine, Hiroshima University, Hiroshima, JPN

**Keywords:** risk factor, hypertension, office blood pressure, morning home blood pressure, early-morning home blood pressure

## Abstract

The patient was a 63-year-old man with a 24-year history of hypertension. During long-term follow-up, when outpatient clinic blood pressure and morning blood pressure are well-regulated, exceptionally elevated early-morning blood pressure does not play a significant role in the development of hypertensive target organ disease or cardiovascular disease.

## Introduction

Various devices are now being widely used for monitoring home blood pressure. There have been improvements in the devices including improvements in usability and reproducibility. Home blood pressure monitoring has several advantages including diagnoses of white-coat hypertension and masked hypertension, marker of target organ damage, prediction of future cardiovascular events, and improvement of adherence with long-term antihypertensive medication treatment and hypertension control rates [1-4]. A combination of measurements of office blood pressure and home blood pressure would enable more specific conclusions concerning the role of measurement of blood pressure in the diagnosis and management of hypertension to be drawn. We present a case in which there were discrepancies between blood pressure measured in the outpatient clinic (office) and that measured at home (early morning and morning) for a long follow-up period.

## Case presentation

A 63-year-old man had a 24-year history of hypertension and had been delivering newspapers for 16 years. He had no history of cardiovascular, cerebrovascular, or renal disease. In April 2000, he was diagnosed with essential hypertension. Secondary forms of hypertension were excluded based on complete history; physical examination; radiological and ultrasound examinations; urinalysis; plasma renin activity; plasma aldosterone and norepinephrine concentrations; serum creatinine, potassium, calcium, and free thyroxine concentrations; and 24-hour urinary excretion of 17-hydroxycorticosteroids, 17-ketogenic steroids, and vanillymandelic acid. In our outpatient clinic, his blood pressure was measured to be 160/96 mmHg. He received the calcium channel blocker amlodipine at a dose of 5 mg per day since April 2000. His home blood pressure was also monitored using a TM2420 device (A&D Co., Tokyo, Japan).

**Figure 1:**
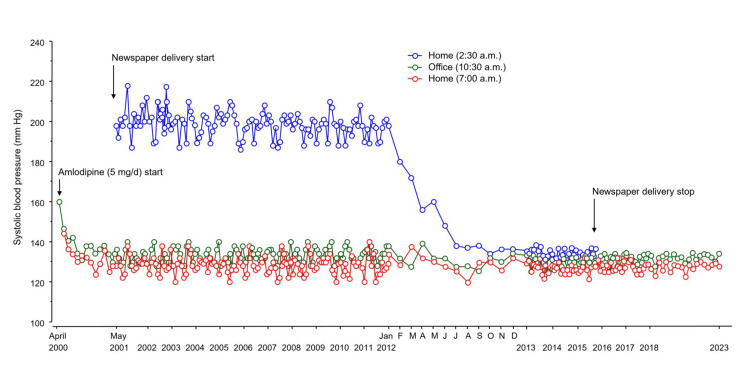
Changes in systolic blood pressures at 2:30 a.m., 10:30 a.m., and 7:00 p.m. during the follow-up period Line graphs show changes in the systolic blood pressures at 2:30 a.m., 10:30 a.m., and 7:00 p.m. during the follow-up period.

After three months, his blood pressure had decreased to 138/76 mmHg and remained in the range of 142/84 to 126/68 mmHg for one year in our outpatient clinic (Figure 1, green and red lines). In May 2001, he started to do a newspaper delivery job. He woke up at 2:00 a.m. and went to bed at 9:00 p.m. every day, and he measured his home blood pressure at 2:30 a.m. before starting his job and at 7:00 a.m. and took amlodipine at 8:30 a.m. after breakfast every day. He maintained this routine for 16 years. His blood pressure was measured at 10:30 a.m. once every month in our outpatient clinic. From May 2001 to January 2012, his early-morning home blood pressure remained high over 180/120 mmHg (Figure 1, blue line). However, his blood pressure in our outpatient clinic and his morning home blood pressure at 7:00 a.m. remained constant from 138/76 to 124/70 mmHg and from 138/78 to 120/70 mmHg, respectively (Figure 1, green line and red line). Interestingly, his early-morning home blood pressure decreased from January 2012 and was 138/78 mmHg in July 2012 and then remained constant from 144/86 to 134/76 mmHg from August 2012 to January 2023 (Figure 1, blue line), while his blood pressure in the outpatient clinic and his morning home blood pressure at 7:00 a.m. were constant (Figure 1, green line and red line). His lifestyle remained unchanged during the follow-up period. Changes in diastolic blood pressure were exactly paralleled by changes in systolic blood pressure. In December 2015, he stopped doing the newspaper delivery job. After stopping his job, he woke up at 6:00 a.m. and went to bed at 11:00 p.m. every day, and he measured his morning home blood pressure at 7:00 a.m. and took amlodipine at 8:30 a.m. after breakfast every day. His blood pressure was measured at 10:30 a.m. once every month in our outpatient clinic. His blood pressure in the outpatient clinic and his home blood pressure at 7:00 a.m. remained constant after stopping the newspaper delivery job (Figure 1, green line and red line). During the follow-up period, there was no cardiovascular, cerebrovascular, or hypertensive target organ disease including chronic kidney disease, left ventricular hypertrophy, and hypertensive retinopathy.

## Discussion

His blood pressure in the outpatient clinic and morning ambulatory blood pressure at 7:00 a.m. were controlled well within an almost normal range after receiving amlodipine and remained constant for 24 years. Home blood pressure at 2:30 a.m. was measured as early-morning blood pressure. He had extremely high early-morning ambulatory blood pressure for about 12 years. However, he had no serious hypertensive target organ disease or cardiovascular disease as well as no albuminuria. Although night-time blood pressure or early-morning blood pressure is measured as one of the components of 24-hour ambulatory blood pressure, there is no information on the changes in the early-morning blood pressure (surge of early-morning blood pressure) in the waking state for a long period of more than 10 years.

It is controversial whether a morning surge in blood pressure contributes to cardiovascular events [5,6]. In the Jackson Heart Study, the relationships between various types of morning blood pressure surge (e.g., sleep-trough type, pre-awakening type, and rising type) measured by 24-hour ambulatory blood pressure monitoring and cardiovascular events over an approximately 14-year follow-up period in blacks were evaluated [7]. Interestingly, each component of blood pressure morning surge was associated with cardiovascular events, while morning blood pressure per se was not associated with cardiovascular events. In this case, although early-morning blood pressure surge was confirmed by a single home blood pressure monitor reading taken 30 minutes after waking, early-morning blood pressure has been measured repeatedly daily for approximately 12 years.

Although office blood pressure has been well-established for hypertension care, office blood pressure is only a cross-sectional measurement of constantly fluctuating blood pressure. With the increasing use of home blood pressure measurement and 24-hour ambulatory blood pressure measurement, there are more opportunities to evaluate blood pressure variability in clinical settings, and the clinical significance of blood pressure variability is attracting attention. However, methods for evaluation of blood pressure variability differ depending on the index, period (e.g., intraday, inter-day, week, months, season, total year, and between visits), and measurement method (office blood pressure, home blood pressure, and 24-hour ambulatory blood pressure), and no unified evaluation standard has been established. To date, various cross-sectional and longitudinal studies have shown an association between blood pressure variability and cardiovascular disease [8,9]. On the other hand, the contribution of blood pressure variability to risk stratification is smaller than that of absolute blood pressure, and in some populations, no association was found between blood pressure variability and cardiovascular disease [10,11]. Although many studies have consistently shown that 24-hour ambulatory blood pressure, especially at night (during sleep), is a better risk marker than office blood pressure for total and cardiovascular mortality [5,12], further studies are needed to clarify the mechanism and determine whether nocturnal blood pressure can be used as an index for blood pressure control.

Recently, studies have focused on the effects of renal denervation on blood pressure in patients with resistance hypertension [13-16]. The results of clinical trials using renal denervation devices on the antihypertensive effect of renal denervation have not been consistent [14-16]. The SYMPLICITY HTN 3 trial [14], the first trial using a renal denervation device of radiofrequency ablation, showed no significant reduction in either 24-hour ambulatory systolic blood pressure or office systolic blood pressure compared to that in the sham group at six months post-treatment. In the SRYRAL HTN-ON MED trial [15] using a technique of radiofrequency ablation and the RADIANCE-HTN trial [16] using a technique of ultrasound ablation, renal denervation showed a significant hypotensive effect as assessed by office systolic blood pressure and 24-hour ambulatory systolic blood pressure for up to six months and two months, respectively, compared to the blood pressure levels in the sham group. In addition to renal denervation, other antihypertensive treatment approaches such as the use of baroreflex activation therapy and cardiac neuromodulation therapy have also been studied. Their antihypertensive effects are still unclear and await investigation using measurements of office blood pressure, home blood pressure, and 24-hour ambulatory blood pressure. Therefore, further research is needed on the purpose of blood pressure change assessment other than risk stratification, what populations should be assessed, and what assessment methods are appropriate.

Although he was not a shift worker, it is well known that shift work is associated with sympathetic nervous system modulation [17,18]. His heart rate and heart rate variability at home and in the outpatient clinic remained constant for 24 years after starting treatment with amlodipine. It is unlikely that sympathetic nervous system activation contributed to the elevation of blood pressure early in the morning. However, we cannot deny the possibility that physiological stress and waking up early in the morning induced sympathetic nervous system activation, leading to elevation of blood pressure. Measurements of biochemical markers of sympathetic nervous system activation would enable more specific conclusions concerning the role of sympathetic nervous system activation in the regulation of early-morning blood pressure to be drawn.

## Conclusions

Although the precise mechanisms by which constantly very high early-morning blood pressure over a period of 12 years suddenly decreased remain unclear, homeostatic compensatory mechanisms may have worked to decrease the stress-induced elevation of blood pressure. During the follow-up period, he received amlodipine at a dose of 5 mg per day and had no serious hypertensive target organ disease or cardiovascular disease under the condition of well-controlled outpatient clinic blood pressure and morning ambulatory blood pressure but not early-morning ambulatory blood pressure. Although it is not conclusive since this is only one case report, under conditions of well-controlled outpatient clinic blood pressure and well-controlled morning blood pressure, extremely high early-morning blood pressure may not contribute to the development of hypertensive target organ disease or cardiovascular disease.
